# Physiologically Based Pharmacokinetic Modelling for Nicotine and Cotinine Clearance in Pregnant Women

**DOI:** 10.3389/fphar.2021.688597

**Published:** 2021-07-20

**Authors:** Basile Amice, Harvey Ho, En Zhang, Chris Bullen

**Affiliations:** ^1^ ENSEEIHT, National Polytechnic Institute of Toulouse, Toulouse, France; ^2^ Auckland Bioengineering Institute, The University of Auckland, Auckland, New Zealand; ^3^ Chongqing Institute for Food and Drug Control, Chongqing, China; ^4^ National Institute for Health Innovation, The University of Auckland, Auckland, New Zealand

**Keywords:** nicotine, cotinine, pregnant women, fetus, PBPK

## Abstract

**Introduction:** Physiologically based pharmacokinetic (PBPK) models for the absorption, disposition, metabolism and excretion (ADME) of nicotine and its major metabolite cotinine in pregnant women (p-PBPK) are rare. The aim of this short research report is to present a p-PBPK model and its simulations for nicotine and cotinine clearance.

**Methods:** The maternal-placental-fetal compartments of the p-PBPK model contain a total of 16 compartments representing major maternal and fetal organs and tissue groups. Qualitative and quantitative data of nicotine and cotinine disposition and clearance have been incorporated into pharmacokinetic parameters.

**Results:** The p-PBPK model reproduced the higher clearance rates of nicotine and cotinine in pregnant women than non-pregnant women. Temporal profiles for their disposition in organs such as the brain were also simulated. Nicotine concentration reaches its maximum value within 2 min after an intravenous injection.

**Conclusion:** The proposed p-PBPK model produces results consistent with available data sources. Further pharmacokinetic experiments are required to calibrate clearance parameters for individual organs, and for the fetus.

## Introduction

Cigarette smoking during pregnancy is associated with many adverse effects, including increased spontaneous abortion, a higher premature delivery rate and lower birth weight (Lambers and Clark, 1996). Clinical and experimental studies on the absorption, disposition, metabolism and excretion (ADME) of nicotine and its major metabolism product, cotinine, in pregnant women have provided important insights (Benowitz and Dempsey, 2004),^,^ such as the significantly higher nicotine and cotinine clearance during pregnancy than post-partum and at different gestation stages (Benowitz and Dempsey, 2004; Dempsey et al., 2002; Taghavi et al., 2018; Benowitz et al., 2006). Possible explanations for this phenomenon include pregnancy-induced metabolism activities for C-oxidation *via* the CYP2A6 and for G-glucuronidation *via* UGT2B10 (Taghavi et al., 2018). Physiological changes during pregnancy may also pay a role, such as the substantially increased renal flow (30–50% higher) and resultant higher renal clearance (Morgan, 1997). To date, however, these findings have not been incorporated into physiologically based pharmacokinetic (PBPK) models for pregnancy (p-PBPK), and specifically for nicotine and cotinine clearance during pregnancy.

PBPK is a mathematical modelling technique for predicting the ADME of drugs in humans. An early PBPK model developed for adult men, not pregnant women, used data from intravenous nicotine infusion experiments to find pharmacokinetic parameters (Robinson et al., 1992). Recent PBPK models for nicotine were also reported, e.g., by Kovar et al. (2020) to simulate nicotine brain tissue concentrations after the use of combustible cigarettes, e-cigarettes, nicotine gums, and nicotine patches, and by Saylor and Zhang (2016) where antibody affinity to nicotine was considered in a PBPK model for nicotine disposition in the brains of rats and humans. Specific to p-PBPK model, Gaohua et al. (2012) used it to investigate the PK profiles of three compounds (caffeine, metoprolol and midazolam) in response to the gestational related activities of three cytochrome P450 enzymes. George et al. (2020) used another p-PBPK model to investigate the dosing adjustment of an antidepressant (sertraline) during pregnancy.

In this study, we take advantage of a generic p-PBPK template (Gentry et al., 2003), where nicotine was used as a representative compound for water soluble, semi-volatile chemicals. However, the model did not provide clearance profiles after nicotine administration but rather changes at different gestation stages. The aim of the current work was to combine the two models, i.e. by Robinson et al. (1992) and Gentry et al. (2003), and to incorporate some recently published data.

## Methods

### Integrated PBPK Model for Nicotine/Cotinine

**FIGURE 1:**
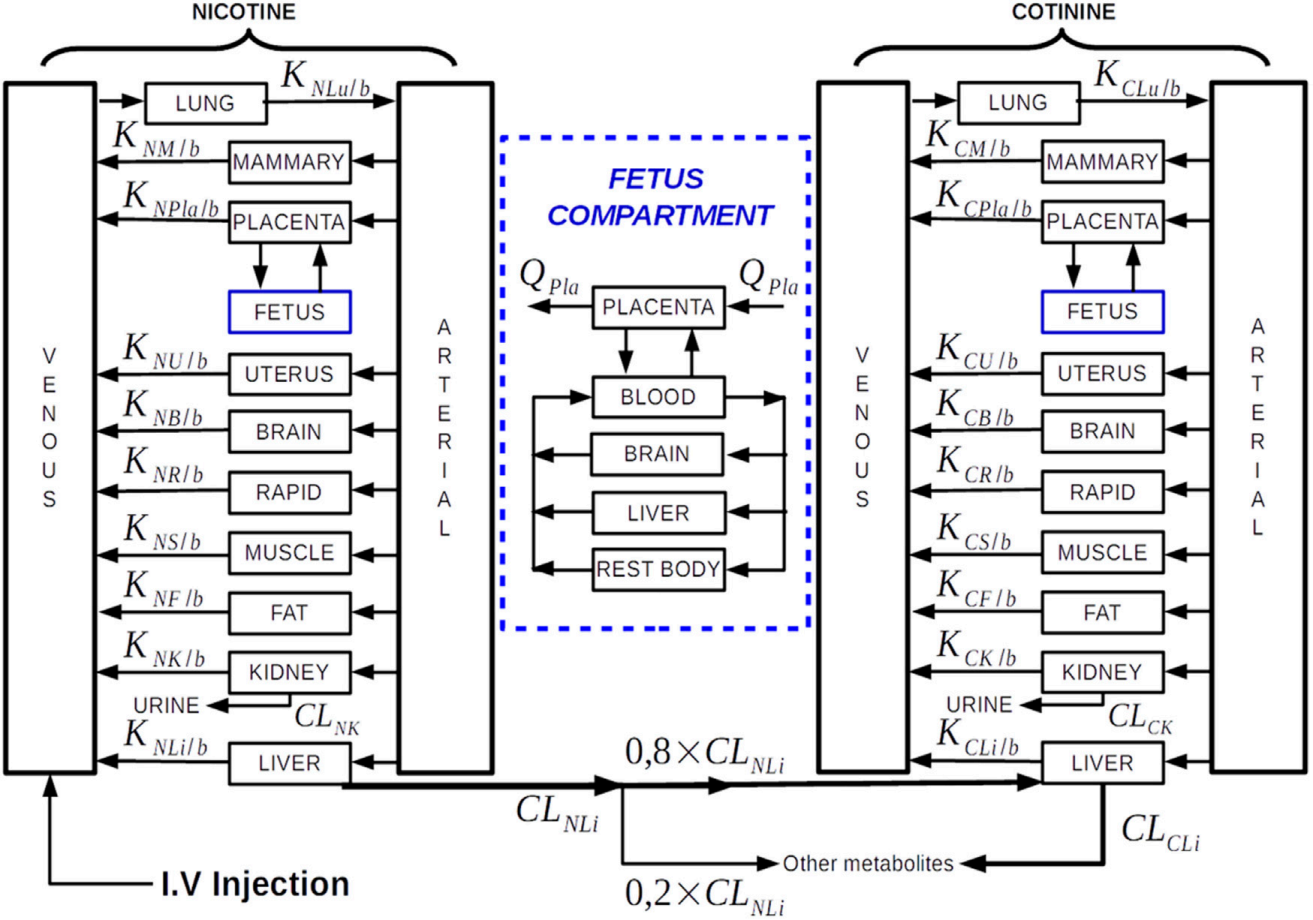
Diagram of the P-PBPK model structure with intravenous nicotine injection as the intake route. The underlying differential equations and parameters are provided in Supplementary Material. Details of the fetus PBPK model are shown inside the blue compartment. Note, the “Rapid” compartment in the diagram corresponding to “Vessel-rich Group” compartment in the PBPK model of Robinson et al. (1992).

We adopted and customised an adult PBPK model consisting of nine compartments for cotinine (COT) and ten for nicotine (NIC), representing key organs and tissues in humans, i.e., the arterial and venous blood, the brain, liver, lung, kidney, rapid (vessel-rich tissues), muscle and fat groups (Robinson et al., 1992) (Figure 1). The NIC and COT models are connected from the liver compartment, representing the biotransformation from nicotine to cotinine *via* CYP2A6 (approximately 80% of nicotine is metabolized into cotinine) (Benowitz et al., 2009). In this way the time course of nicotine and cotinine concentrations can be simulated simultaneously. Furthermore, we added an extra brain compartment to simulate the quick uptake of nicotine in the brain (10–20 s after cigarette smoking) (Benowitz et al., 2009).

A significant difference between our PBPK model and the model of (Robinson et al., 1992) is the updated renal and hepatic clearance rates since women have a higher nicotine/cotinine clearance (Dempsey et al., 2002) (Curvall et al., 1990). The parameters for NIC/COT hepatic and renal clearance, including those estimated for pregnant women, are shown in Table 1.

**TABLE 1:** Nicotine and cotinine hepatic and renal clearance parameters used in the model.

	Parameter for men Curvall et al. (1990), ^a^	Parameters for women Dempsey et al. (2002), ^b^	Parameters for pregnant women, estimated from Dempsey et al. (2002), Taghavi et al. (2018), ^b^
Nicotine hepatic clearance	277.14	16.2	26.6
Nicotine renal clearance	0.6198	0.7	0.3
Cotinine hepatic clearance	6.3635	0.5	1.2
Cotinine renal clearance	0.0248	0.2	0.3

a Unit: ml/h/kg.

b Unit: ml/min/kg.

In addition to the hepatic and renal clearance changes, the clearance rate in the muscle compartment is also updated so that the nicotine concentration in muscle is similar to that in the plasma (Benowitz et al., 2009).

### P-PBPK Model Construction

The p-PBPK model has been constructed with extra compartments: the mammary, uterus, placenta and fetus compartments (Figure 1). We adopted the p-PBPK template which has a similar NIC-COT compartmental structure to (Gentry et al., 2003). We also used some physiological parameters in this template, as documented in the Supplementary Material. In the fetus compartment, in addition to the blood, liver and rest of the body compartments, a brain compartment is added to investigate the nicotine distribution to the fetal brain. Concerning the methods of nicotine administration, three pathways have been implemented including intravenous injection, cigarette smoking and oral dosing.

New clearance rates have been estimated to simulate the accelerated clearance rates for nicotine and cotinine in pregnant women, which are about 60 and 140% higher respectively than non-pregnant women (Dempsey et al., 2002; Taghavi et al., 2018). Corresponding changes to the hepatic and cotinine renal clearances are shown in Table 1.

In the p-PBPK model of Gentry et al. (2003), the transfer of drugs across the placenta barrier is modelled in a diffusion-limited equation. However, since the fetal nicotine level is 15% higher than the maternal side, yet the fetal cotinine level is lower than the maternal level (Lambers and Clark, 1996), an influx-efflux model is used to simulate the nicotine/cotinine transfer in the placenta. Different stages of gestation are also incorporated through adjustments of organ/tissue volumes, blood flow supply based on the generic equations (Abduljalil et al., 2012; Brown et al., 1997).

### Parameters of the p-PBPK Model

Overall, our model consists of two sets of parameters. The first set of parameters describe the physiological properties in each compartment including the volume, blood flow rate; while the second set of parameters define drug-specific parameters including tissue-to-blood partition coefficients, metabolic and clearance rates. The second set, i.e. nicotine/cotinine related parameters including their respective data sources are provided in Table 1. The first set i.e., physiological parameters are provide in the Supplementary Material.

There are total 32 differential equations for the p-PBPK model shown in Figure 1. The equations are not listed here but provided in the Supplementary Material for interested reader’s reference. The program was implemented in Matlab, with ODE45 as the differential equation solver. To run the programme, the gestation stage (in months) and body weight (in kg) need to be provided. In this work we used gestation week 30 and body weight 73 kg as the parameters, which can be altered by the user. Furthermore, the body weight is related to the gestation week, which has also been implemented in our model (Sharma et al., 2018).

### Validation of the Model

Published plasma NIC/COT concentration data that was used to validate the PBPK model in (Robinson et al., 1992), were employed to validate the non-pregnant woman model. Specifically, the plasma levels of cotinine for four non-smoking subjects after cotinine infusion (0.67 mg/min for 30 min), as reported by De Schepper et al. (1987), and the plasma NIC/COT concentration after nicotine infusion (10 µg/min for 60 min) in six non-smoking subject, as reported by (Curvall et al., 1990), were used to compare with our model simulations.

The studies on plasma NIC/COT levels in pregnant women are very rare. However, there are reports that describe qualitatively some pharmacokinetic features of NIC/COT in pregnant women. For instance (Lambers and Clark, 1986), pointed out that the nicotine level in plasma at the fetal side was about 15% higher than that at the maternal side. These data have been used indirectly in our model for parameter optimisation.

## Results

### Baseline PBPK Model

**FIGURE 2:**
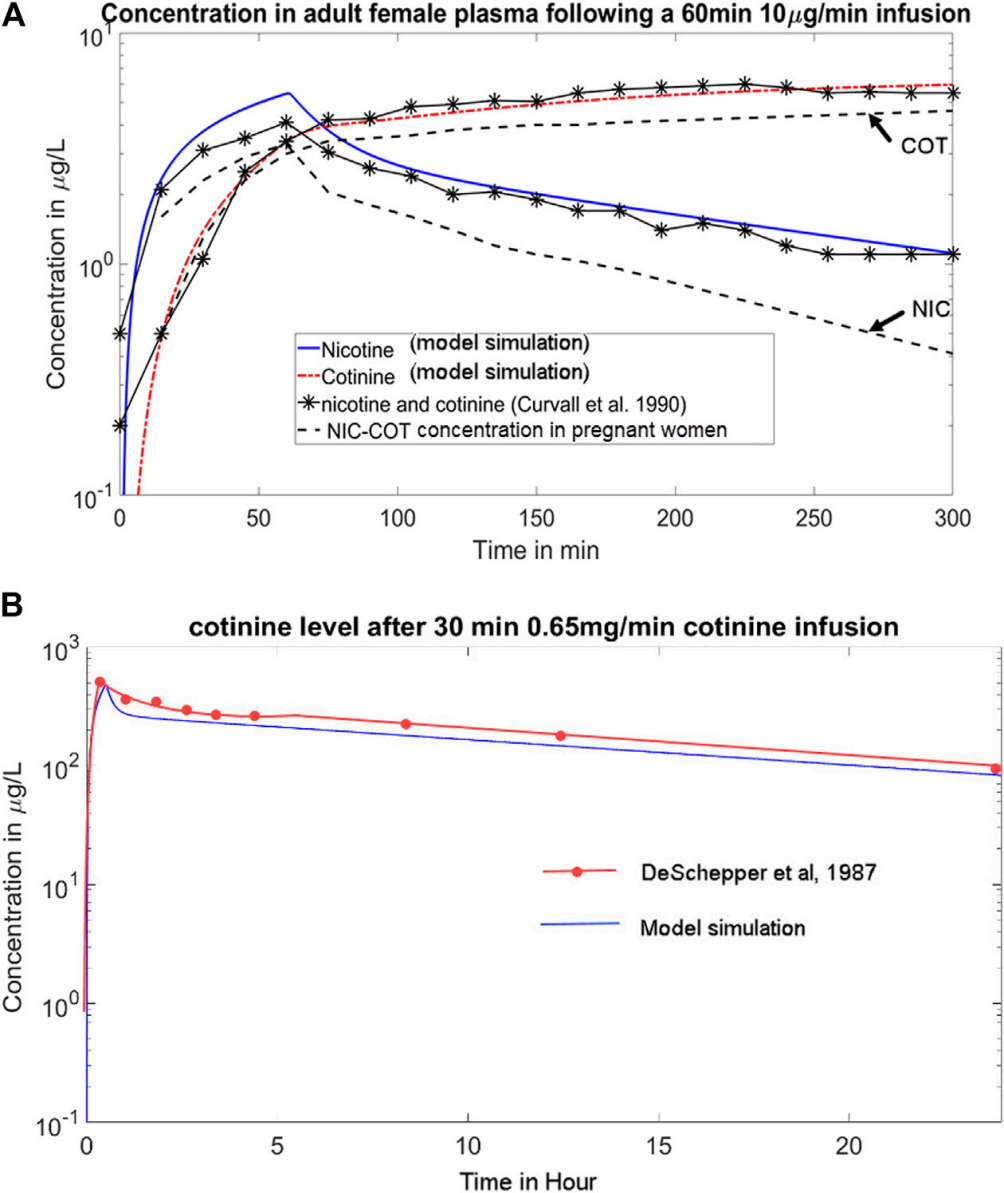
**(A)** Simulations of the time course of the plasma concentrations of NIC/COT following a 60 min 10 μg/min intravenous infusion of nicotine, per data from Curvall et al. (1990). The blue solid line and red dotted line represent the NIC-COT concentration in adult men, matching experimental data (solid lines with asterisks), which the dashed lines represent plasma concentration of NIC-COT in pregnant women showing higher clearance rates; **(B)** Simulation of the cotinine level after 30 min of 0.65 mg/min continine infusion, per data from De Schepper et al. (1987). The blue line represents model simulation.

At first, we calibrated the adult PBPK model with published nicotine/cotinine PBPK model for man (Robinson et al., 1992), which may be used as a proxy for non-pregnant women. The simulations in (Robinson et al., 1992) contain several dose and infusion scenarios, which we chose two regimens to investigate: 1) intravenous nicotine infusion of 10 µg/min for 60 min (Curvall et al., 1990); and 2) intravenous infusion of cotinine of 0.67 mg/min for 30 min (De Schepper et al., 1987). The body weight of the adult women was set as 70 kg, following the adult body weight configuration in (Robinson et al., 1992). Figure 2 shows the time course of nicotine and cotinine over 5 h. The plasma nicotine concentration reaches the peak value after about 1 h, and then gradually decreases. Its half-life (∼3 h) is much shorter than that of cotinine (∼16 h), in accordance with the half-life data reported in literature (2 h for nicotine vs. 16.6 h for cotinine) (Dempsey et al., 2002).

The time course of the concentration of nicotine and cotinine in plasma (blue and red lines respectively) resulting from our model match closely (within 5% of deviation) with the pharmacokinetic data reported by Curvall et al. (1990). In addition, the literature reported 22 ± 7.2 µg of unchanged nicotine and 16.1 ± 3.8 µg of cotinine in urine 5 h after the infusion (Curvall et al., 1990), while our model predicted 28.8 µg of nicotine and 15.3 µg of cotinine in urine, consistent with the literature.

### p-PBPK Model

The evolvement of nicotine and cotinine centration profiles in pregnant women was simulated. The gestation stage was set as week 30, and the body weight of pregnant woman as 73 kg. At this stage of fetal development the fetal liver has limited nicotine and cotinine metabolism and clearance capacity (Benowitz et al., 2009). Since data related to fetal clearance was not available, assumptions were made that the fetal clearance efficiency was only 20% of maternal hepatic clearance for both nicotine and cotinine. With these assumptions, the plasma nicotine and cotinine profiles are shown as dashed lines in Figure 1. As can be seen, the nicotine and cotinine concentrations in the pregnant women model are lower than the adult non-pregnant woman model, reflecting higher clearance rates. This is more pronounced in nicotine (∼70% lower) than in cotinine (∼30% lower) at 150 min.

**FIGURE 3:**
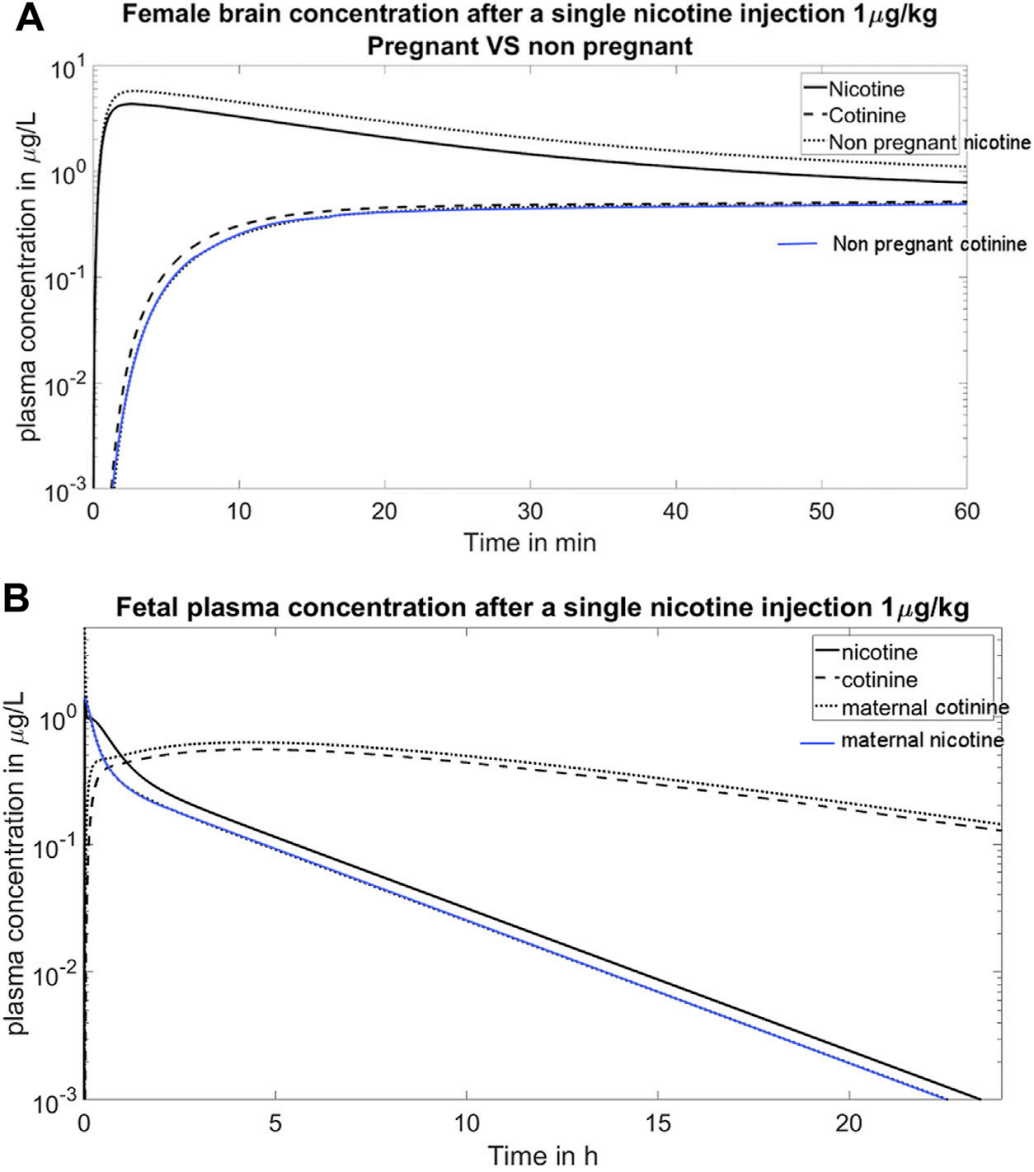
**(A)** Simulation of the time course of the concentration of nicotine and cotinine in the maternal brain. The nicotine enters brain quickly and reaches its peak value within 2 min. This figure also shows the higher clearance of nicotine in pregnant women than in non-pregnant women; **(B)** Simulation of fetal plasma concentration after a single nicotine injection 1 μg/kg. The simulation reproduces the clinical observation that the fetal nicotine level is higher than the maternal nicotine level, yet the fetal cotinine level is lower than the maternal level (Lambers and Clark, 1996).

After a puff of cigarette smoking, the nicotine concentration in the brain increases rapidly (Benowitz and Dempsey, 2004). This fast entrance phenomenon also occurs with intravenous injection, as shown in the simulation of Figure 3, where the nicotine concentration in the brain reaches its maximum value within 2 min. Also shown in Figure 3 is the higher nicotine clearance in the brain during pregnancy, as the concentration profile of nicotine is lower in pregnant women than non-pregnant women.

## Discussion

Approximately 14% of United States women continue to smoke after becoming pregnant (Taghavi et al., 2018), and an estimated 32% of women who are Māori (the indigenous people of New Zealand) smoke during pregnancy (Humphrey et al., 2016). Nicotine replacement therapy (NRT) has been used for smoking cessation assistance during pregnancy in the forms of nicotine gums, transdermal administration and patches (Benowitz and Dempsey, 2004). Still, the pharmacokinetic profiles of nicotine in individual organs of pregnant women, in particular in fetus, remain poorly understood. Most experiments provide measurement data from plasma or urine samples as it is difficult to obtain tissue measurements *in vivo*. This is even more the case when drug clearance in fetus needs to be investigated, as blood samples are taken from the umbilical vein/artery only at the time of delivery. The motivation of the work was to develop an *in silico* p-PBPK model for the prediction of nicotine and cotinine clearance, and to provide an initial computational platform for incorporating new data and/or for evaluating new hypotheses. Another motivation of the model was to incorporate the simulation results into graphic animations for educational purposes. A science-based, visual tool could aid public health workers to explain the pharmacokinetics of nicotine/cotinine in a more understandable manner.

The current model combines two previous PBPK models with an updated set of nicotine and cotinine specific parameters to reflect our updated knowledge of their clearance in pregnant women (Dempsey et al., 2002; Taghavi et al., 2018). Only a subset of results are presented in this short report due to scarcity of *in vivo* or *in vitro* data to compare with. However, we found that the partition coefficients in the fetal model and its clearance did not have great impacts on the maternal model. Rather, the physiological changes over different gestation stages could exert significant influence on the nicotine clearance. Another important finding was that placental absorption/clearance plays an important role in mediating the overall nicotine/cotinine kinetics in fetus. This effect was previously simplified as a first order diffusion effects (Gentry et al., 2003), which was not sufficient to explain the transportation of nicotine and cotinine across the placenta barrier. For example, to transport cotinine from the fetus to the mother, where the cotinine level is higher, a more sophisticated model than the passive diffusion model is required.

It should be stressed that even though the current p-PBPK model, with a non-trivial set of 32 differential equations, is still highly simplified due to the complexity of drug disposition and clearance in the maternal-placental-fetal compartments. For example, the hepatic and renal clearance parameters for nicotine are gestational age dependent, which in the current model are fixed (corresponding to gestational week 30). Likewise are the partition coefficients, or unbound fractions, which may alter during different stages of gestation. Further investigation into individual nicotine metabolism pathways *via* CYPs and UGTs would require a novel model involving nonlinear metabolism kinetic terms. Such a model should be tested for the liver compartment at first for optimal parameters, before applying it to a larger PBPK model. We refer the interested reader to such an individual enzyme-oriented metabolism model of acetaminophen for reference (Means and Ho, 2019).

It worth noting that the placental barrier plays an important role in the drug transfer between maternal and fetal circulations. While our p-PBPK model has additional influx-efflux terms for the placenta compartment, it is not sufficient to describe the complex transport mechanism of nicotine/cotinine in placenta. Specifically, various transporters play a critical role in the apical and basolateral membrane of trophoblasts, where their mediation kinetics warrants a separate study. We refer the interested reader for an excellent review on this topic by Dallmann et al. (2019).

In this report we only presented simulation results where nicotine intake was *via* intravenous infusion, because the hour-based PK data for verifying simulation results were available (Curvall et al., 1990) (De Schepper et al., 1987). However, it should be noted that the most common route for nicotine intake is *via* smoking, and the oral intake e.g., by chewing nicotine gums is the most common for NRT (Oncken et al., 1996). However, since serum cotinine data were taken after days’ of gum use (Oncken et al., 1996), they cannot be used to verify hour-based PK profile simulations.

Due to the difficulty of obtaining data in humans, an extension of the current model is to adapt it to animals, and to compare the results with published data from animal models (Craig et al., 2014). Still, due to the significant differences of nicotine metabolism between different species (Hukkanen et al., 2005), cautions must be taken to extrapolate the model between species.

Concerning application of the p-PBPK framework to other drugs, physiological aspect of the model, i.e., blood flow/volume to individual organs/tissues may still be applicable, or with only minor adjustments required. However, drug-specific parameters, such as the hepatic/renal clearance, the drug’s volume of distribution and partition coefficient, must be re-instilled. Moreover, efforts should be made to obtain first-hand pharmacokinetic data where the elimination kinetics are independently informed.

Another extension of the work would be to apply the model to population pharmacokinetics analysis. Several approaches could be employed towards that direction. For example, a Latin Hypercube Sampling analysis could be performed where model parameters are perturbed around their nominal values simultaneously (Zhang et al., 2020). By observing the statistical distribution of pharmacokinetic profiles of NIC/COT in a population, we could determine the influence of a parameter on system dynamics i.e., the sensitivity of a model versus its parameters.

In summary, there are many future extension possibilities, such as longitudinal studies, enzyme activities, hepatic/renal clearance changes, could be incorporated into the current prototype model. While adding these many features would be interesting, adding these features in the model demands multidisciplinary collaborations on data collection, physiological interpretation, and model refining.

## Conclusion

A p-PBPK model has been developed for nicotine and cotinine disposition and clearance. The model has reproduced some key features of ADME in pregnant women. More data are required to calibrate the parameters in the model.

## Data Availability

The datasets presented in this study can be found in online repositories. The names of the repository/repositories and accession number(s) can be found in the article/Supplementary Material.
