# Supplementary material for: Physiologically Based Pharmacokinetic Modelling for Nicotine and Cotinine Clearance in Pregnant Women
Source: Front Pharmacol. 2021 Jul 20;12:688597. doi: 10.3389/fphar.2021.688597 (PMC8329445; doi:10.3389/fphar.2021.688597)
Supplement: Supplementary file 1 [file DataSheet1.PDF]

MATERNAL NICOTINE

ARTERIAL

$$\frac{d}{dt}\left(C_{NA}\right)=\frac{1}{V_A}Q_{blood}\left(\frac{C_{NLu}}{K_{NLu/b}}-C_{NA}\right) \tag{1}$$

FAT

$$\frac{d}{dt}\left(C_{NF}\right)=\frac{1}{V_F}Q_F\left(C_{NA}-\frac{C_{NF}}{K_{NF/b}}\right) \tag{2}$$

SLOWLY PERFUSED TISSUES

$$\frac{d}{dt}\left(C_{NS}\right)=\frac{1}{V_S}Q_S\left(C_{NA}-\frac{C_{NS}}{K_{NS/b}}\right) \tag{3}$$

RAPIDLY PERFUSED TISSUES

$$\frac{d}{dt}\left(C_{NR}\right)=\frac{1}{V_R}Q_R\left(C_{NA}-\frac{C_{NR}}{K_{NR/b}}\right) \tag{4}$$

BRAIN

$$\frac{d}{dt}\left(C_{NB}\right)=\frac{1}{V_B}Q_B\left(C_{NA}-\frac{C_{NB}}{K_{NB/b}}\right) \tag{5}$$

MAMMARY

$$\frac{d}{dt}\left(C_{NM}\right)=\frac{1}{V_M}Q_M\left(C_{NA}-\frac{C_{NM}}{K_{NM/b}}\right) \tag{6}$$

UTERUS

$$\frac{d}{dt}\left(C_{NU}\right)=\frac{1}{V_U}Q_U\left(C_{NA}-\frac{C_{NU}}{K_{NU/b}}\right) \tag{7}$$

KIDNEY

$$\frac{d}{dt}\left(C_{NK}\right)=\frac{1}{V_K}\left(Q_K\left(C_{NA}-\frac{C_{NK}}{K_{NK/b}}\right)-fu_N CL_{NK}\frac{C_{NK}}{K_{NK/b}}\right) \tag{8}$$

LIVER

$$\frac{d}{dt}\left(C_{NLi}\right)=\frac{1}{V_{Li}}\left(Q_{Li}\left(C_{NA}-\frac{C_{NLi}}{K_{NLi/b}}\right)-fu_N CL_{NLi}\frac{C_{NLi}}{K_{NLi/b}}\right) \tag{9}$$

LUNG

$$\frac{d}{dt}\left(C_{NLu}\right)=\frac{1}{V_{Lu}}Q_{blood}\left(C_{NVe}-\frac{C_{NLu}}{K_{NLu/b}}\right) \tag{10}$$

VENOUS

$$\frac{d}{dt}\left(C_{NVe}\right)=\frac{1}{V_{Ve}}\left(\begin{array}{r}Dose_{I.V}+Q_F\frac{C_{NF}}{K_{NF/b}}+Q_S\frac{C_{NS}}{K_{NS/b}}+Q_R\frac{C_{NR}}{K_{NR/b}}+Q_B\frac{C_{NB}}{K_{NB/b}}+\\ Q_M\frac{C_{NM}}{K_{NM/b}}+Q_U\frac{C_{NU}}{K_{NU/b}}+Q_K\frac{C_{NK}}{K_{NK/b}}+Q_{Li}\frac{C_{NLi}}{K_{NLi/b}}-Q_{blood}C_{NVe}\end{array}\right) \tag{11}$$

MATERNAL COTININE

ARTERIAL

$$\frac{d}{dt}\left(C_{CA}\right) = \frac{1}{V_A} Q_{blood}\left(\frac{C_{CLu}}{K_{CLu/b}} - C_{CA}\right) \tag{12}$$

FAT

$$\frac{d}{dt}\left(C_{CF}\right) = \frac{1}{V_F} Q_F\left(C_{CA} - \frac{C_{CF}}{K_{CF/b}}\right) \tag{13}$$

SLOWLY PERFUSED TISSUES

$$\frac{d}{dt}\left(C_{CS}\right) = \frac{1}{V_S} Q_S\left(C_{CA} - \frac{C_{CS}}{K_{CS/b}}\right) \tag{14}$$

RAPIDLY PERFUSED TISSUES

$$\frac{d}{dt}\left(C_{CR}\right) = \frac{1}{V_R} Q_R\left(C_{CA} - \frac{C_{CR}}{K_{CR/b}}\right) \tag{15}$$

BRAIN

$$\frac{d}{dt}\left(C_{CB}\right) = \frac{1}{V_B} Q_B\left(C_{CA} - \frac{C_{CB}}{K_{CB/b}}\right) \tag{16}$$

MAMMARY

$$\frac{d}{dt}\left(C_{CM}\right) = \frac{1}{V_M} Q_M\left(C_{CA} - \frac{C_{CM}}{K_{CM/b}}\right) \tag{17}$$

UTERUS

$$\frac{d}{dt}\left(C_{CU}\right) = \frac{1}{V_U} Q_U\left(C_{CA} - \frac{C_{CU}}{K_{CU/b}}\right) \tag{18}$$

KIDNEY

$$\frac{d}{dt}\left(C_{CK}\right) = \frac{1}{V_K}\left(Q_K\left(C_{CA} - \frac{C_{CK}}{K_{CK/b}}\right) - fu_C CL_{CK}\frac{C_{CK}}{K_{CK/b}}\right) \tag{19}$$

LIVER

$$\frac{d}{dt}\left(C_{CLi}\right) = \frac{1}{V_{Li}}\left(Q_{Li}\left(C_{CA} - \frac{C_{CLi}}{K_{CLi/b}}\right) + 0.8 fu_N CL_{NLi}\frac{C_{NLi}}{K_{NLi/b}} - fu_C CL_{C-met}\frac{C_{CLi}}{K_{CLi/b}}\right) \tag{20}$$

LUNG

$$\frac{d}{dt}\left(C_{CLu}\right) = \frac{1}{V_{Lu}} Q_{blood}\left(C_{CVe} - \frac{C_{CLu}}{K_{CLu/b}}\right) \tag{21}$$

VENOUS

$$\frac{d}{dt}\left(C_{CVe}\right) = \frac{1}{V_{Ve}}\begin{pmatrix} Q_F\frac{C_{CF}}{K_{CF/b}} + Q_S\frac{C_{CS}}{K_{CS/b}} + Q_R\frac{C_{CR}}{K_{CR/b}} + Q_B\frac{C_{CB}}{K_{CB/b}} \\ +Q_K\frac{C_{CK}}{K_{CK/b}} + Q_{Li}\frac{C_{CLi}}{K_{CLi/b}} - Q_{blood}C_{CVe} \end{pmatrix} \tag{22}$$

PLACENTA

NICOTINE

$$\frac{d}{dt}(C_{NPla}) = \frac{1}{V_{Pla}}\left(Q_{PLa}\left(C_{NA} - \frac{C_{NPla}}{K_{NPla/b}}\right) - PAF\left(\frac{V_{maxN}}{K_{mN} + C_{NPla}}\frac{C_{NPla}}{K_{NPla/b}} - C_{NFetBld}\right)\right) \quad (23)$$

COTININE

$$\frac{d}{dt}(C_{CPla}) = \frac{1}{V_{Pla}}\left(Q_{PLa}\left(C_{NA} - \frac{C_{CPla}}{K_{CPla/b}}\right) - PAF\left(\frac{V_{maxC}}{K_{mC} + C_{CPla}}\frac{C_{CPla}}{K_{CPla/b}} - C_{CFetBld}\right)\right) \quad (24)$$

PAF:the interstitial fluid exchange in trophoblasts between the maternal and fetal sides.

Comments

1.15 and 0.88 refers to the Lambers and Clark article(1996). Indeed it is mentionned in this paper that the fetal blood concentration in nicotine is 15% higher than in mother blood. For the cotinine it is mentionned that the fetal blood concentration is 88% of the mother concentration.

FETUS

NICOTINE

LIVER

$$\frac{d}{dt}\left(C_{NFetLi}\right) = \frac{1}{V_{FetLi}} Q_{FetLi} \left(C_{NFetBld} - \frac{C_{NFetLi}}{K_{NFetLi/b}} \right) \tag{25}$$

BRAIN

$$\frac{d}{dt}\left(C_{NFetB}\right) = \frac{1}{V_{FetB}} Q_{FetB} \left(C_{NFetBld} - \frac{C_{NFetB}}{K_{NFetB/b}} \right) \tag{26}$$

REST OF BODY

$$\frac{d}{dt}\left(C_{NFetRB}\right) = \frac{1}{V_{FetRB}} Q_{FetRB} \left(C_{NFetBld} - \frac{C_{NFetRB}}{K_{NFetRB/b}} \right) \tag{27}$$

BLOOD

$$\frac{d}{dt}\left(C_{NFetBld}\right) = \frac{1}{V_{FetBld}} \left(\begin{array}{r} Q_{FetLi}\dfrac{C_{NFetLi}}{K_{NFetLi/b}} + Q_{FetB}\dfrac{C_{NFetB}}{K_{NFetB/b}} + Q_{FetRB}\dfrac{C_{NFetRB}}{K_{NFetRB/b}} \\ +PAF\left(\dfrac{V_{maxN}}{K_{mN} + C_{NPla}} \dfrac{C_{NPla}}{K_{NPla/b}} - C_{NFetBld} \right) - Q_{FetBld} C_{NFetBld} \end{array} \right) \tag{28}$$

COTININE

LIVER

$$\frac{d}{dt}\left(C_{CFetLi}\right) = \frac{1}{V_{FetLi}} Q_{FetLi} \left(C_{CFetBld} - \frac{C_{CFetLi}}{K_{CFetLi/b}} \right) \tag{29}$$

BRAIN

$$\frac{d}{dt}\left(C_{CFetB}\right) = \frac{1}{V_{FetB}} Q_{FetB} \left(C_{CFetBld} - \frac{C_{CFetB}}{K_{CFetB/b}} \right) \tag{30}$$

REST OF BODY

$$\frac{d}{dt}\left(C_{CFetRB}\right) = \frac{1}{V_{FetRB}} Q_{FetRB} \left(C_{CFetBld} - \frac{C_{CFetRB}}{K_{CFetRB/b}} \right) \tag{31}$$

BLOOD

$$\frac{d}{dt}\left(C_{CFetBld}\right) = \frac{1}{V_{FetBld}} \left(\begin{array}{r} +Q_{FetLi}\dfrac{C_{CFetLi}}{K_{CFetLi/b}} + Q_{FetB}\dfrac{C_{CFetB}}{K_{CFetB/b}} + Q_{FetRB}\dfrac{C_{CFetRB}}{K_{CFetRB/b}} \\ +PAF\left(\dfrac{V_{maxC}}{K_{mC} + C_{CPla}} \dfrac{C_{CPla}}{K_{CPla/b}} - C_{CFetBld} \right) - Q_{FetBld} C_{CFetBld} \end{array} \right) \tag{32}$$

NOMENCLATURE

Concentrations C

$$C_{\boxed{a}\boxed{T}}$$

-a=N for nicotine concentration and a=C for cotinine concentration.
-T designate the compartement, cf Table 1.

Examples :
-C_{NLi} = Nicotine concentration in the maternal liver
-C_{NFetB} = Nicotine concentration in the fetal brain
-C_{CFetB} = Cotinine concentration in the fetal brain

Partition coefficients K

$$K_{\boxed{a}\boxed{T}/b}$$

-a=N for nicotine blood partition coefficients and a=C for cotinine blood partition coefficients.
-T designate the compartement, cf Table 1.

$$K_{\boxed{a}\boxed{T}/b} = \frac{C_{tissue(T)}}{C_{Venous(Ve)}}$$

Examples :
-$K_{NLi/b}$ = Nicotine blood partition coefficient of the maternal liver
-$K_{NFetB/b}$ = Nicotine blood partition coefficient of the fetal brain
-$K_{CFetB/b}$ = Cotinine blood partition coefficient of the brain

Clearance Coefficient CL

$$CL_{\boxed{a}\boxed{T}/b}$$

-a=N for nicotine blood partition coefficients and a=C for cotinine blood partition coefficients.
-T designate the compartement, (Li or K) cf Table 1.

Blood flows Q

$$Q_{\boxed{T}}$$

-T designate the compartement, cf Table 1.
-Q_{blood} designate the total blood flow/cardiac outpout.

Examples :
-Q_{Li} = Blood flow to the maternal liver
-Q_{FetB} = Blood flow to the fetal brain

Organs volumes V

$$V_{\boxed{T}}$$

-T designate the compartement, cf Table 1.

Examples :
-V_{Li} = Volume of the maternal liver
-V_{FetB} = Volume of to the fetal brain

Absorbtion parameters
PAF

Table 1: Compartment designation

**T**	**compartment**
A	Arterial
F	Fat[(1)]
S	Slowly perfused tissues
R	Rapidly perfused tissues
B	Brain
M	Mammary[(1)]
U	Uterus[(1)]
K	Kidney
Li	Liver
Lu	Lung
Ve	Venous
Pla	Placenta[(1)]
FetLi	Fetus liver[(1)]
FetB	Fetus brain[(1)]
FetRB	Fetus rest of the body[(1)]
FetBld	Fetus blood[(1)]

[(1)] blood flow rate and volume depend over time

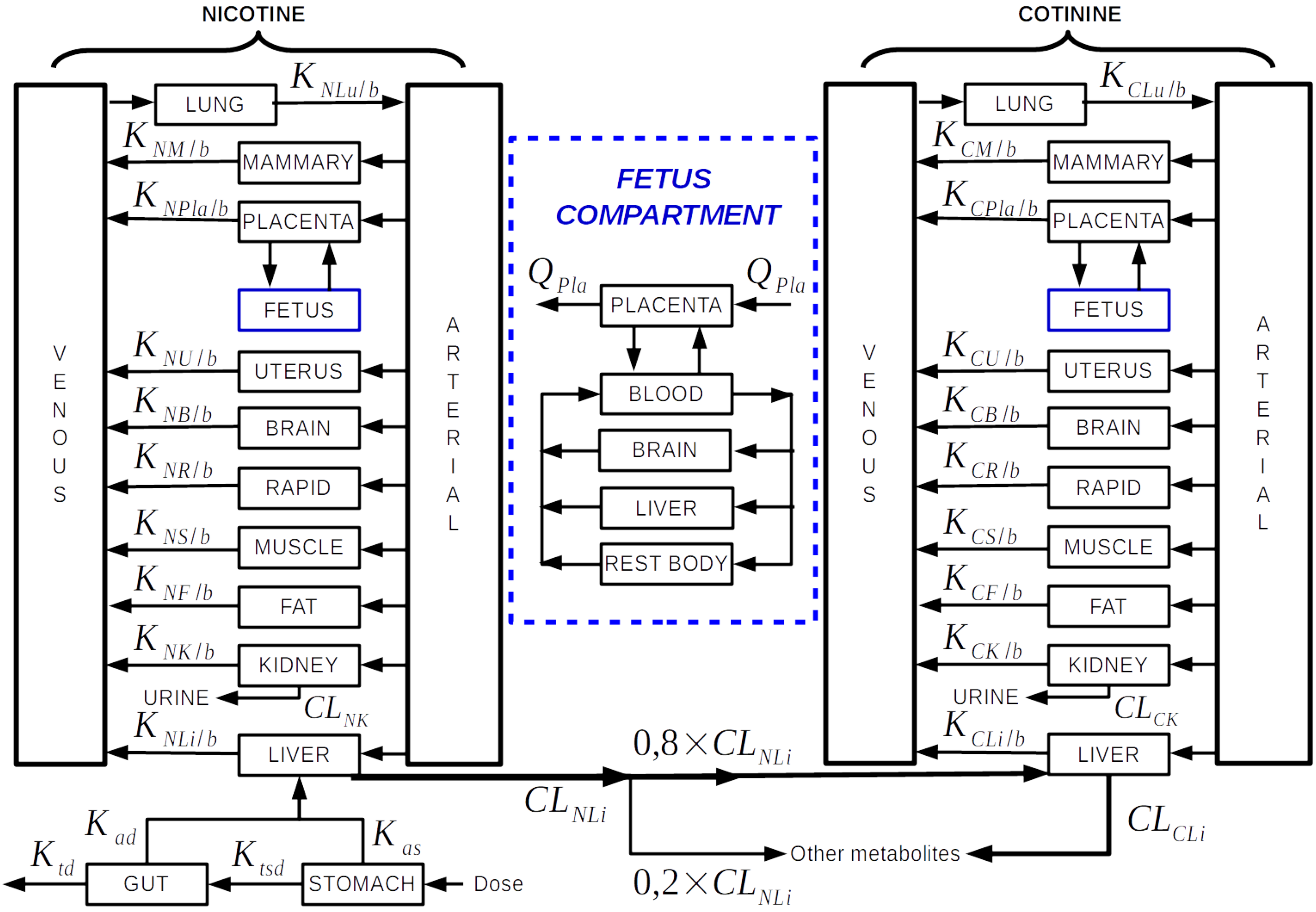

Figure 1: Nicotine and Cotinine pathways

$$V_{mammary}\left(GD\right) = V_{mammary-init} + BW_{init} * 0.0065 * e^{-7.44486 * e^{-0.000678 * GD * 24}}$$

$$V_{uterus}\left(GD\right) = V_{uterus-init} + BW_{init} * 0.02 * e^{-4.715669973 * e^{-0.000376 * GD * 24}}$$

$$V_{fat}\left(GD\right) = V_{fat-init} + BW_{init} * 0.09 * e^{-12.909955862 * e^{-0.000797 * GD * 24}}$$

$$V_{fetus}\left(GD\right) = 3.779 * e^{-16.08 * e^{-5.67 * 10^{-4} * GD * 24}}$$

$$V_{placenta}\left(GD\right) = 0.85 * e^{-9.434 * e^{-5.23 * 10^{-4} * GD * 24}}$$

$$BW = BW_{init} + V_{mammary} - V_{mammary-init} + V_{uterus} - V_{uterus-init} + V_{fat} - V_{fat-init} + V_{fetus} + V_{placenta}$$

$$V_{bloodfetus} = F_{blood-fetus} * V_{fetus}$$

$F_{blood-fetus} = 0.0085$

$$Q_{mammary} = Q_{mammary-init} * \frac{V_{mammary}}{V_{mammary-init}}$$

$$Q_{uterus} = Q_{uterus-init} * \frac{V_{uterus}}{V_{uterus-init}}$$

$$Q_{fat} = Q_{fat-init} * \frac{V_{fat}}{V_{fat-init}}$$

$$Q_{placenta} = FQ_{placenta} * V_{placenta}$$

$FQ_{placenta} = 58.4L/h/kg$

$$Q_{blood-fetus} = FQ_{blood-fetus} * V_{fetus}$$

$FQ_{blood-fetus} = L/h/kg$

$$Q_{blood} = Q_{blood-init} + Q_{mammary} - Q_{mammary-init} + Q_{uterus} - Q_{uterus-init} + Q_{fat} - Q_{fat-init} + Q_{placenta}$$
