# Supplementary material for: Physiologically Based Pharmacokinetic Modelling for Nicotine and Cotinine Clearance in Pregnant Women
Source: Front Pharmacol. 2021 Jul 20;12:688597. doi: 10.3389/fphar.2021.688597 (PMC8329445; doi:10.3389/fphar.2021.688597)
Supplement: Supplementary file 2 [file DataSheet2.docx]

**Supplementary File**

**Physiologically based pharmacokinetic modelling for nicotine and cotinine clearance in pregnant women**

B. Amice, H. Ho, E. Zhang, C. Bullen

**S.1: Diagram of the p-PBPK model**

The PBPK model in Robinson 1992 (Robinson et al., 1992) consists of ten compartments for nicotine (NIC model) and nine for cotinine (COT model), representing the arterial and venous blood, the liver, lung, kidney, gut, vessel-rich, muscle and fat groups. A significant difference between our model and the Robinson 1992 model is the updated renal and hepatic clearance coefficients to resolve the problem of overestimation of nicotine/cotinine clearance in. We adopted the parameters from (Dempsey et al., 2002):

- Nicotine hepatic clearance 277.14 $(k\frac{g^{0.25}}{h})$→ 16.2 $(ml/min/kg)$ (Dempsey et al., 2002)
- Nicotine renal clearance 0.6198 $(L/h/kg^{0.75})$→ 0.7 $(ml/min/kg)$ (Dempsey et al., 2002)
- Cotinine hepatic clearance 6.3635 $(k\frac{g^{0.25}}{h})$→ 0.5 $(ml/min/kg)$ (Dempsey et al., 2002)
- Cotinine renal clearance 0.0248$(k\frac{g^{0.25}}{h})$→ 0.2 $(ml/min/kg)$ (Dempsey et al., 2002)

The p-PBPK model is based on a generic template, (Gentry et al., 2003) which contains 13 compartments for different organs and tissue groups of pregnant women. The minor modification in the diagram is the addition of “brain” compartments to the maternal and fetal model. The major difference is the incorporation of nicotine and cotinine-specific parameters such as their partition coefficients and clearance rates, (Robinson et al., 1992) which were verified with experimental data available at that time. The diagram of the p-PBPK model is shown in Fig. S1. The parameters of individual compartments are shown in S2.


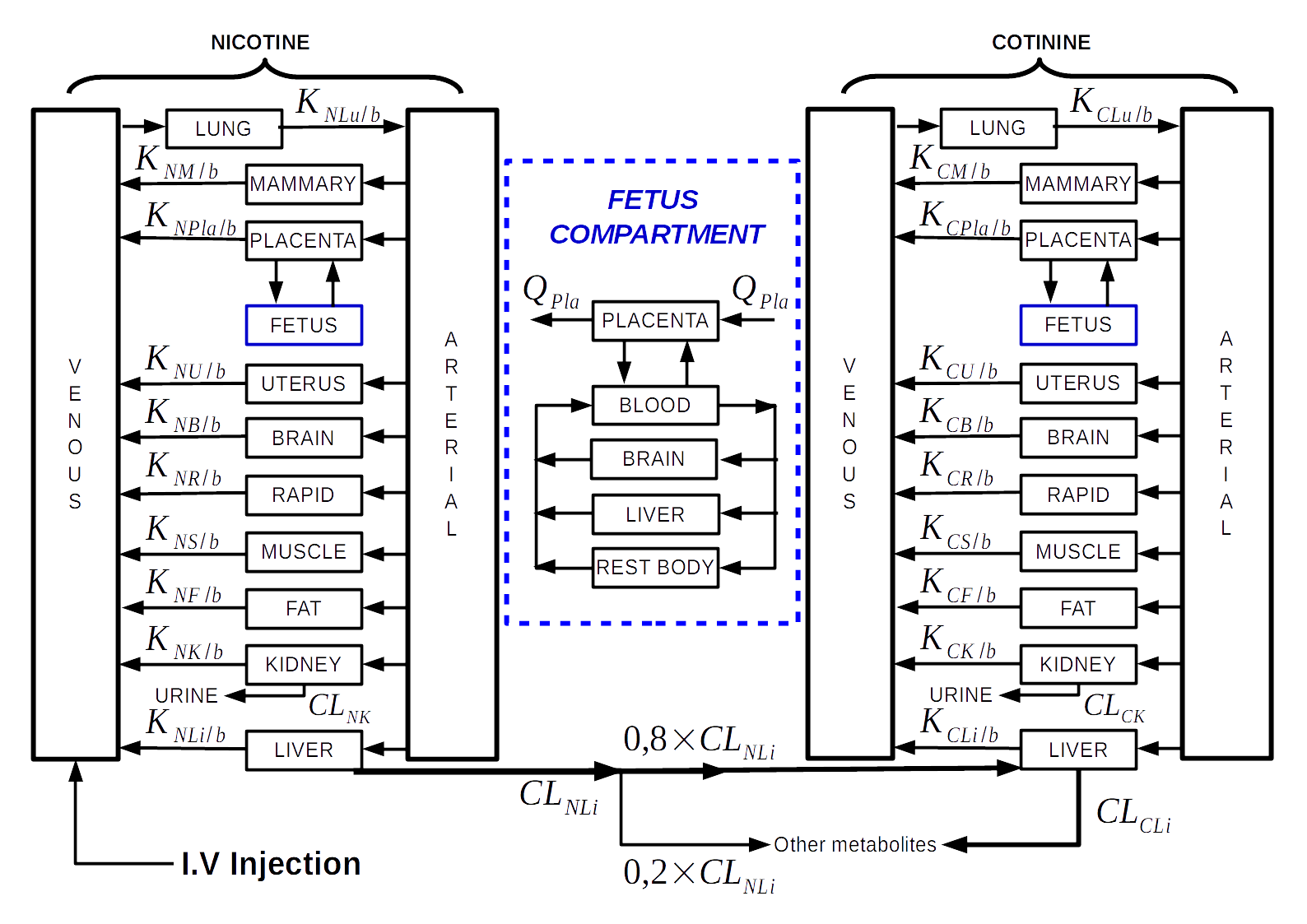


*Fig. S1: The structure of the P-PBPK model. Details of the fetus PBPK model are shown inside the blue compartment.*

Significant changes occur during pregnancy for the clearance of nicotine and cotinine. (Dempsey et al., 2002) The clearance for nicotine and cotinine was significantly higher (60 and 140% respectively) for pregnant women. Corresponding changes made to the equation system include:

- Nicotine hepatic clearance 16.2 $(k\frac{g^{0.25}}{h})$→ 26.6 $(ml/min/kg)$ (Dempsey et al., 2002)
- Nicotine renal clearance 0.7 $(ml/min/kg)$→ 0.3 $(ml/min/kg)$ (Dempsey et al., 2002)
- Cotinine hepatic clearance 0.5$(ml/min/kg)$ → 1.2 $(ml/min/kg)$ (Dempsey et al., 2002)
- Cotinine renal clearance 0.2$(ml/min/kg)$→ 0.3 $(ml/min/kg)$ (Dempsey et al., 2002)

**S2. Parameters of the p-PBPK model**

The nicotine/cotinine model in (Robinson et al., 1992) is for adults. However, the model of (Robinson et al., 1992) overestimated the clearance of nicotine and cotinine. This discrepancy however is offset by the fact that pregnant women do have higher clearance rates for nicotine/cotinine than non-pregnant women.

*Table S.1: Compartment specific physiological parameters for adults*

| Organ/tissue | Volume fraction to body weight  Ftissue | Data source | Blood flows fraction to cardiac output  FQtissue | Data source |
| --- | --- | --- | --- | --- |
| Arterial | 0.02 | (Robinson et al., 1992) | 1 | (Robinson et al., 1992) |
| Fat | 0.187 | (Brown et al., 1997) | 0.052 | (Brown et al., 1997) |
| Slow | 0.536 | (Clewell et al., 2001) | 0.188 | (Clewell et al., 2001) |
| Rapid | 0.032 | (Clewell et al., 2001) | 0.216 | (Clewell et al., 2001) |
| Brain | 0.021 | (Brown et al., 1997) | 0.117 | (Brown et al., 1997) |
| Kidney | 0.004 | (Brown et al., 1997) | 0.177 | (Brown et al., 1997) |
| Liver | 0.026 | (Brown et al., 1997) | 0.25 | (Brown et al., 1997) |
| Lung | 0.008 | (Brown et al., 1997) | 1 | (Brown et al., 1997) |
| Venous | 0.0571 | (Robinson et al., 1992) | 1 | (Robinson et al., 1992) |
| Vit (infusion compartment) | 0.0214 | (Robinson et al., 1992) |  |  |
| Cardiac output | | | | |
| Cardiac output fraction to body weight | | FCO ( L/h/kg0.75 ) | 15.126 | (Robinson et al., 1992) |

The parameters used in the calculation of the volumes and blood flow rates during the pregnancy are detailed below:

*Table S.2: Parameters for pregnancy compartments.*

| Organ/tissue | Volume fraction to body weight  Ftissue | Blood flows fraction to cardiac output  FQtissue | Nicotine partition coefficient | Cotinine partition coefficient | Source |
| --- | --- | --- | --- | --- | --- |
| Mammary | 0.0062 | 0.027 | 2.5 | 1.5 | (Gentry et al., 2003) |
| Uterus | 0.0014 | 0.0062 | 3 | 1.5 | (Gentry et al., 2003) |
| Rapid | 0.0244 | 0.1828 | 3 | 1.5 | (Clewell et al., 2001) |
| Fetus liver | 0.04 | 0.25 same as mother | 9 same as mother | 2 same as mother | ICRP 2002 |
| Fetus brain | 0.011 | 0.117 same as mother | 3 same as mother | 1 same as mother | ICRP 2002 |
| Fetus blood | 0.085 | / | 1 | 1 | (Clewell et al., 2001) |
| Fetus rest body | 0.9530 | 0.6330 | 3 rapidly perfused assumption because of fetal dev | 1.5 rapidly perfused assumption because of fetal dev |  |
| Placental kinetics | | | | | |
| metabolism | nicotine | | cotinine | | source |
| Vmax (nmol/min/kgof placenta) | 1 | | 1 | | Optimize* |
| Km (nmol/L) | 1000 | | 1000 | | Optimize* |
| Transfer rates | | | | |  |
| Mother → Fetus | | Fetus → Mother | |  |  |
| Kt1(L/h) | 1500 | Kt2(L/h) | 1000 |  | Optimize* |
| Kt3(L/h) | 880 | Kt4(L/h) | 1000 |  | Optimize* |

*The *in silico* experiments for the placenta model here was to reproduce the NIC/COT observations reported in Lambers & Clark (1996). Kt1, Kt2, Kt3 and Kt4 represent the influx/efflux parameters for NIC/COT respectively, corresponding to a single exchange interface between maternal/fetal circulations. The parameters presented here were our try-and-error approach to find numerical values that generate the expected pharmacokinetics of NIC/COT in maternal/fetal blood.

A better model would have three compartments: Maternal capillaries <-> trophoblast <-> fetal capillaries, and also incorporate the role transporters play here. This work deserves a separate study in its own right.

*Table S.3: Drug-specific parameters for nicotine/cotinine*

| Clearance | Nicotine | Cotinine | Source |
| --- | --- | --- | --- |
| Hepatic(ml/min/kg) | 16.2 (KFC) | 0.5 (KFC1) | (Dempsey et al., 2002) |
| Renal(ml/min/kg) | 0.7 | 0.2 | (Dempsey et al., 2002) |
| Unbound fraction | 0.95 | 0.974 | (Robinson et al., 1992) |
| Blood/plasma ratio | 0.8 | 0.88 | (Robinson et al., 1992) |
| Tissue/Blood partition coefficients | | | |
|  |  |  |  |
| Fat/Blood | 1 | 0.5 | (Robinson et al., 1992) |
| Slow/Blood | 2.5 | 0.85 | (Robinson et al., 1992) |
| Rapid/Blood | 3 | 1.5 | (Robinson et al., 1992) |
| Brain/Blood | 3 | 1.5 | (Gentry et al., 2003) |
| Kidney/Blood | 15 | 2 | (Robinson et al., 1992) |
| Liver/Blood | 9 | 2 | (Robinson et al., 1992) |
| Lung/Blood | 2 | 1 | (Robinson et al., 1992) |
| Infusion kinetics | | | |
| Transfer rate (VIT→ lung) | Kvit ( min-1 ) | 4.1 | (Robinson et al., 1992) |

**S3. Differential equations for the p-PBPK model**

The differential equation system for the p_PBPK model, which contains total 32 equations, is attached as a separate file “p_PBPK_equations.pdf”

**References**

Brown, R. P., Delp, M. D., Lindstedt, S. L., Rhomberg, L. R., and Beliles, R. P. (1997). Physiological Parameter Values for Physiologically Based Pharmacokinetic Models. *Toxicol Ind Health* 13, 407–484. doi:10.1177/074823379701300401.

Clewell, H. J., Gentry, P. R., Gearhart, J. M., Covington, T. R., Banton, M. I., and Andersen, M. E. (2001). Development of a Physiologically Based Pharmacokinetic Model of Isopropanol and Its Metabolite Acetone. *Toxicol Sci* 63, 160–172. doi:10.1093/toxsci/63.2.160.

Dempsey, D., Jacob, P., and Benowitz, N. L. (2002). Accelerated Metabolism of Nicotine and Cotinine in Pregnant Smokers. *J Pharmacol Exp Ther* 301, 594–598. doi:10.1124/jpet.301.2.594.

Gentry, P. R., Covington, T. R., and Clewell, H. J. (2003). Evaluation of the potential impact of pharmacokinetic differences on tissue dosimetry in offspring during pregnancy and lactation. *Regulatory Toxicology and Pharmacology* 38, 1–16. doi:10.1016/S0273-2300(03)00047-3.

Lambers, D. S., and Clark, K. E. (1996). The maternal and fetal physiologic effects of nicotine. *Seminars in Perinatology* 20, 115–126. doi:10.1016/S0146-0005(96)80079-6.

Robinson, D. E., Balter, N. J., and Schwartz, S. L. (1992). A physiologically based pharmacokinetic model for nicotine and cotinine in man. *Journal of Pharmacokinetics and Biopharmaceutics* 20, 591–609. doi:10.1007/BF01064421.
